# A Case Report of Two Kala-azar Cases in China Diagnosed by Metagenomic Next-Generation Sequencing

**DOI:** 10.3389/fmed.2022.922894

**Published:** 2022-08-30

**Authors:** Hongguang Gao, Jing Wang, Shu Zhang, Tian Li

**Affiliations:** ^1^Department of Emergency Medicine, West China Hospital, Sichuan University, Chengdu, China; ^2^Precision Medicine Key Laboratory of Sichuan Province, West China Hospital, Sichuan University, Chengdu, China; ^3^School of Basic Medicine, Fourth Military Medical University, Xi'an, China

**Keywords:** kala-azar, *Leishmania*, mNGS, fever, diagnosis

## Abstract

**Background:**

Leishmaniasis being a local disease, as kala-azar this particular form is a visceral form. It is transmitted by sandflies, and is a parasitic disease involving the reticuloendothelial system of mononuclear macrophages. Due to its poor prognosis and high fatality rate, the fatality rate of patients without effective treatment can exceed 95%. Thereby, early diagnosis and treatment can significantly improve its prognosis. The metagenomic next-generation sequencing (mNGS) has the advantage of being able to find pathogens that cannot be detected by traditional methods. More importantly, it can conduct nucleic acid detection of pathogens covering a wide range in a short time. For infectious diseases like kala-azar, which is clinically complicated and difficult, mNGS detection provides a basis for accurate etiological diagnosis.

**Case Report:**

We report 2 cases of kala-azar in West China Hospital, Chengdu, China. The first case is a 47-year-old male patient who had recurrent fever for 4 months, accompanied by reduction of red blood cell, white blood cell, and blood platelet. He was detected by mNGS and clinically diagnosed as kala-azar (*Leishmania* detection), finally died of multiple organ failure. The second patient was a 15-year-old male who had fever for more than 10 days. He was detected by mNGS and clinically diagnosed as kala-azar (*Leishmania* detection). He recovered and discharged quickly after treatment with sodium stibogluconate.

**Conclusion:**

Efforts should be made to improve early etiological diagnosis in order to improve patient prognosis. mNGS detection is beneficial to the diagnosis and treatment of infectious diseases with unknown causes in the early stage of emergency treatment.

## Background

*Visceral leishmaniasis* (VL) is caused by *Leishmania* protozoa. The parasite is spread by sandflies. It is commonly known as kala-azar (1). Kala-azar is a parasitic disease involving the reticuloendothelial system of mononuclear macrophages (2). At present, the incidence of leishmaniasis has been significantly reduced in China, whereas some provinces in northwest/southwest China are still leishmaniasis endemic areas (3). Visceral leishmaniasis remains endemic in the Mediterranean basin, East Africa, the Indian subcontinent, Rahman et al. (4). Visceral leishmaniasis is considered to be an emerging disease in Europe (5). The clinical manifestations of kala-azar are complex, characterized by long-term irregular fever, progressive splenomegaly, emaciation, anemia, pancytopenia, and increased plasma globulin (1). Due to its poor prognosis and high fatality rate, the fatality rate of patients without effective treatment can exceed 95% (2). Thereby, early diagnosis and treatment can significantly improve its prognosis.

Clinical emergency doctors lack sufficient understanding and attention to kala-azar, and insufficient epidemiological history data have been collected so far. Therefore, it is difficult to distinguish kala-azar from hematological and other infectious diseases; and a misdiagnose or missed diagnosis can easily occur. At present, the clinical diagnosis of kala-azar is mainly achieved through bone marrow smear, histopathological biopsy etiology, or rK39 immunochromatographic strip test, among which the discovery of *Leishmania* parasite in bone marrow, lymph nodes, spleen, and other tissues is considered the golden criterion of diagnosis (3). However, the histopathological examination requires a long operation period, which might delay diagnosis and treatment. The rK39 immunochromatographic strip detection is an unconventional detection method that can only be performed in specific institutions. The PCR detection has high specificity and can improve the detection level of *Leishmania*, but it has not been widely used in China (6). Metagenomic second-generation sequencing is unbiased, high-coverage, accurate, and reliable; thus, it has been gradually applied to parasitic infectious diseases (7). Metagenomic sequencing can effectively detect parasite sequences that cannot be detected by conventional methods, and has been widely applied to clinical practice.

In the emergency department of our hospital, two male patients with fever of unknown cause were not clearly diagnosed by conventional etiological tests. The mNGS has the advantage of being able to find pathogens that cannot be detected by traditional methods. More importantly, it can conduct nucleic acid detection of pathogens covering a wide range in a short time (8). The diagnosis was confirmed by the next-generation metagenomic sequencing (mNGS) detection of *Leishmania* protozoa in the early emergency treatment, providing suggestions for accurate diagnosis and treatment of clinical infectious diseases at an early stage.

## Metagenomic Next-Generation Sequencing Data Analysis and Case Reports

### Metagenomic Next-Generation Sequencing Methods and Data Analysis

A total of 5–10 ml of whole blood was collected from each patient and stored in an EDTA tube at 4°C. Samples were centrifuged within 1 h after collection for 10 min at 1,600 × *g* at 4°C. Plasma samples were transferred to 1.5-ml microcentrifuge tubes and stored at −80°C until DNA extraction. Nucleic acids were extracted from 0.3 ml plasma, and DNA libraries were established by shearing, end repair, A-tailing, adapter ligation, and PCR amplification. DNA libraries were denatured and circularized. DNA nanoballs were generated with the ssDNA circle by rolling circle replication. Finally, sequencing was performed on a BGI MGISEQ-2000 platform in the SE50 format for 12 h. The sequencing raw data were preprocessed by removing contained adapter sequences, reads with high N base and low-complexity reads based on in-house software. Human reads and reads of internal reference were computationally extracted using Burrows–Wheeler Alignment (9). Subsequently, the remaining data were identified through BWA alignment to the pathogen sequence database (PMDB). Finally, pathogens were annotated with all classified mapped reads. The total reads number and the reads number of internal reference for each sample were counted to control the sample quality, and the draft reports were generated automatically by interpretation logic. Draft reports were reviewed and revised by infectious disease physicians according to patients' clinical symptoms and other laboratory test results to generate the final reports.

### Case I of a 47-Year-Old Male Patient

Patient 1: Male, 47 years old, technical staff. He came to our hospital for emergency treatment in December 28, 2020 due to “recurrent fever for 4 months with aggravation accompanied with diarrhea for 1 month.” About 4 months ago, the patient suddenly developed fever without an evident cause, with the highest temperature of 39.9°C. The fever usually occurred in the afternoon and dropped to normal temperature after several hours, accompanied by headache, muscle pain, and nausea. The patient took “antipyretics and artemisinin” orally; however, the temperature did not come down significantly. He was referred to multiple hospitals for treatment but ended with no definite diagnosis.

**Figure 1:**
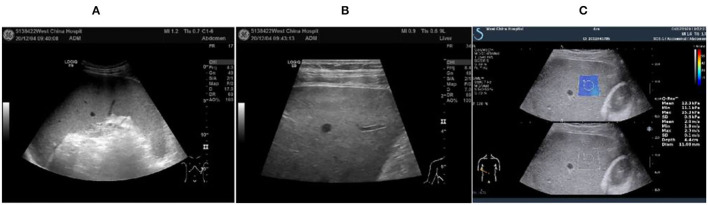
Abdominal ultrasound image of the 47-year-old at 2020.12.04. **(A)** Intercostal spleen thickness was ~7.0 cm and parenchymal echo was uniform; **(B)** Hepatomegaly with liver parenchymal damage; **(C)** Liver hardness measurement: 12.3 kpa.

**Figure 2:**
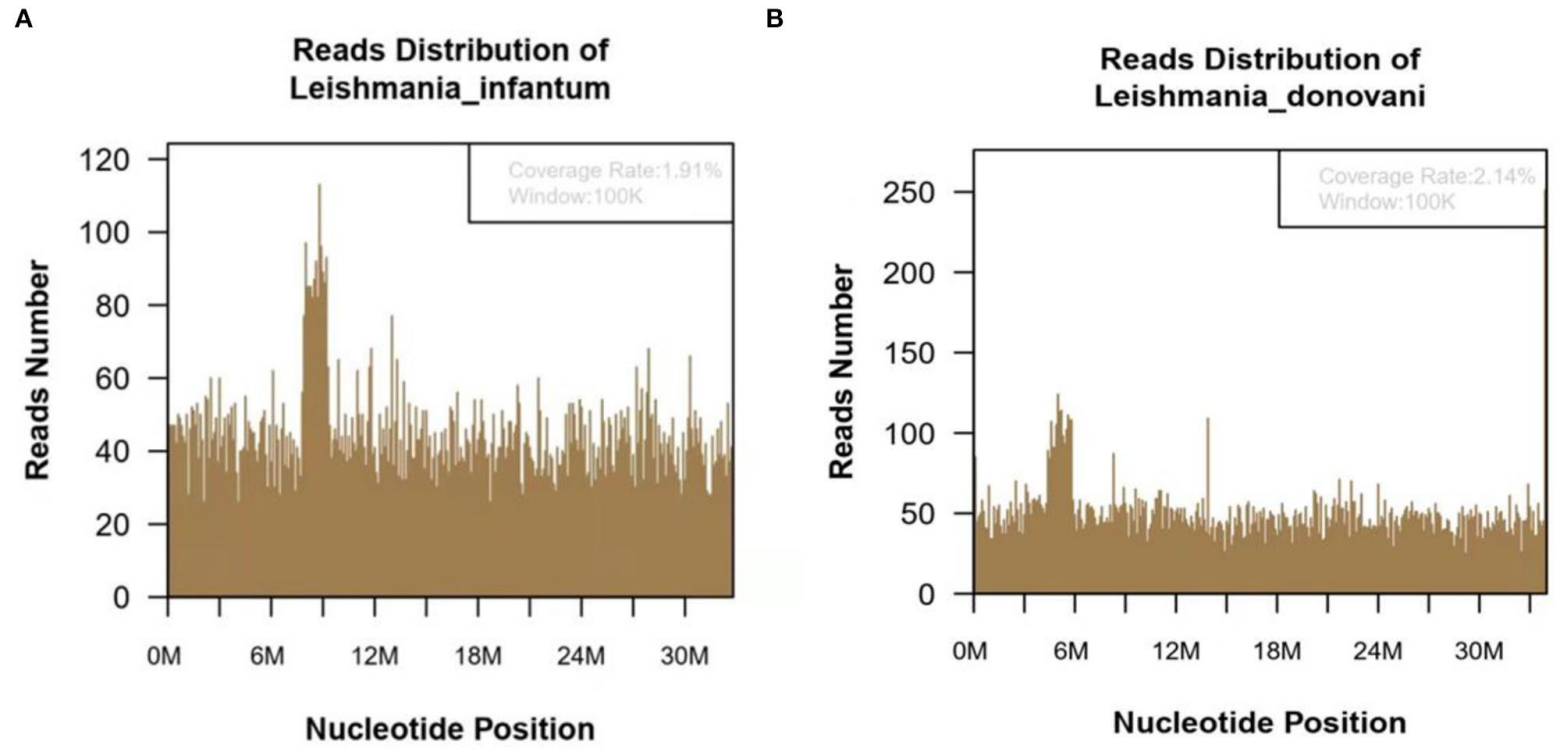
The metagenomic next-generation sequencing (mNGS) of blood sample of 47-year-old kala-azar patient. **(A)** mNGS result showed 118,783 microorganism sequence reads and 14,812 Leishmania Infantum reads, which accounted for 1.91% (626118/32803598) of the whole genome. **(B)** mNGS of blood sample showed a total of 118,783 microorganism sequence reads, and 17,299 reads of *Leishmania Donovani*, accounting for 2.14% (726792/34012130) of the whole genome.

In October 2020, he was discharged from a local hospital after his body temperature returned to normal. One month after discharge, the fever reoccurred with a temperature of 39°C, which appeared as a persistent high fever and endured significantly longer than before. The high fever was accompanied by diarrhea for more than 10 times per day, which appeared as yellow liquid stool. The past medical history: 6 years ago, the patient was diagnosed with malaria in Africa and was treated with oral Quinine. Examination results after admission: Novel Coronavirus nucleic acid test: negative, blood cell analysis (five categories): Hb 83 g/L, Plt count 16 × 10^9^/L, WBC count 2.82 × 10^9^/L, NE percentage 84.0%, lymphocyte percentage 8.0%, and procalcitonin 0.87 ng/ml. Blood culture: methicillin-resistant coagulase-negative Staphylococci. The patient was hypersensitive to all quinolones, such as moxifloxacin. The cryptococcus antigen test, fungal G test, and GM test were negative. The schistosoma japonicum IgG antibody, echinococcus granulosus antibody, liver fluke antibody test, plasmodium antigen test, and blood smear test were all negative. Other tests such as hepatitis A virus and hepatitis B virus were also negative. Autoimmune liver, alpha fetoprotein (AFP), and carcinoembryonic antigen were negative. Abdominal ultrasound showed enlarged liver and spleen (Figure 1). Chest and plain abdominal CT: cirrhosis, splenomegaly, portal hypertension with collateral circulation, and peritoneal effusion. The following diagnosis was considered: sepsis, fever of undetermined origin (FUO): infection? The tumor? Child–Pugh B stage of decompensated cirrhosis. An emergency mNGS blood test was performed to diagnose the fever with unknown cause. Even after being treated with moxifloxacin anti-infective therapy, the patient continued to suffer from recurrent fever. The blood mNGS test results on the 4th day of admission detected *Leishmania infantum* and *Leishmania donovani* (Figure 2). Suspected diagnosis: kala-azar. One day later, the Centers for Disease Control and Prevention performed an rK39 antibody test and the result appeared positive; therefore, the clinical diagnosis of kala-azar was made, and the patient was subjected to sodium stibogluconate (SSG) treatment. According to the treatment standard of 90–130 mg/kg, a total of 9000 mg SSG was used (the maximum dose was given according to the weight of kg) and then stopped. The SSG was divided into 8 days, 600 mg on the first day and 1,200 mg/d after the first day. During the treatment, the patient persistently showed anemia, thrombocytopenia, and coagulation dysfunction, so repeated transfusion therapy was administered. After 3 days of the treatment, the patient's temperature decreased and the fever never returned. However, after 10 days of sodium stibogluconate treatment, the patient developed decreased blood pressure and multiple organ failure (progressive deterioration of coagulation, renal function, and liver function and continued thrombocytopenia). Septic shock was considered, and active anti-infection, fluid replenishment, and non-invasive ventilation were arranged. The patient's condition was not alleviated, and his family refused to adopt emergency treatment measures, such as endotracheal intubation, chest compressions, and continuous renal replacement therapy (CRRT). The patient died 18 days after admission.

### Case II of a 15-Year-Old Male Patient

**Figure 3:**
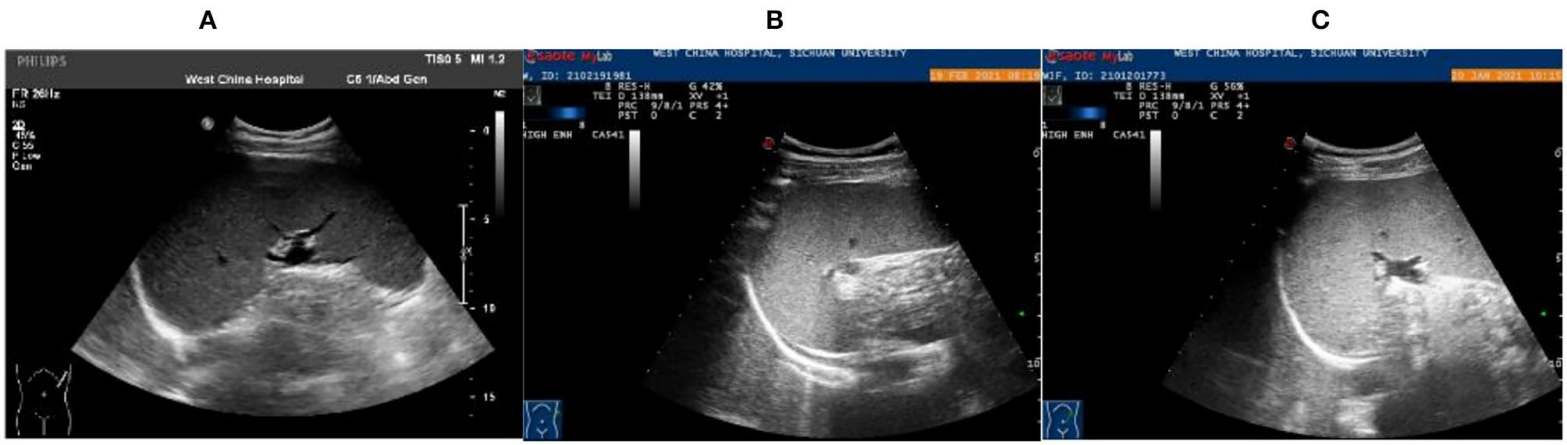
Abdominal color ultrasound of 15-year-old patient**. (A)** Examination results on the first day of admission showed that the intercostal spleen thickness was approximately 6.6 cm and parenchymal echo was uniform, indicating splenomegaly. **(B)** On the 14th day of admission, the spleen was retracted, and the thickness of the spleen was about 5.1 cm. **(C)** Follow-up 1 month after discharge, the spleen was retracted, and the thickness was about 4.5 cm.

**Figure 4:**
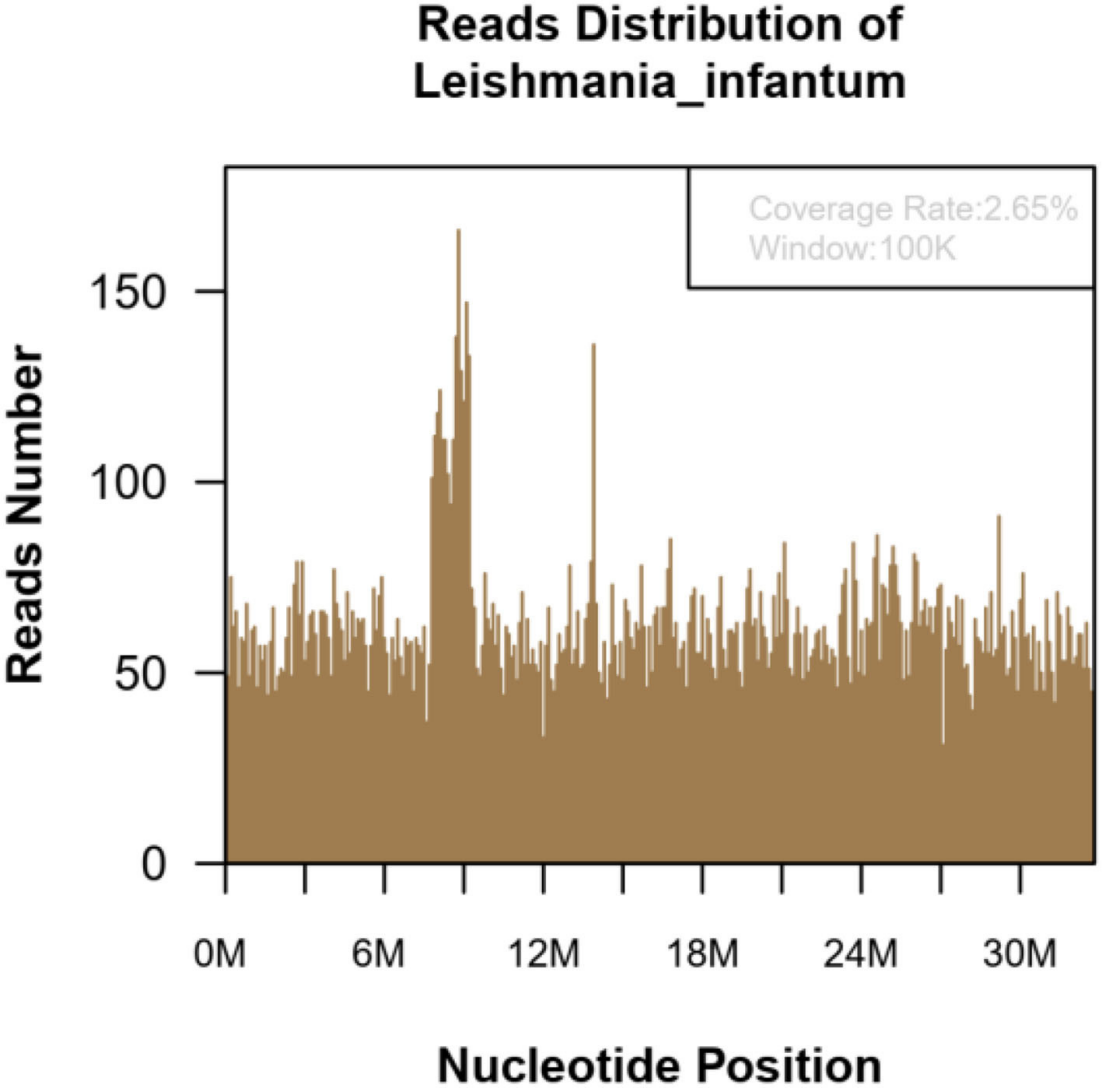
The mNGS of the blood sample of 15-year-old patient. Result showed 139,030 microorganism sequence reads and 20,629 Leishmania Infantum reads, which accounted for 2.65% (869709/32803598) of the whole genome.

Patient 2: Male, 15 years old, a middle-school student. He lived in Chongqing for a long time and was admitted to the emergency fever clinic chamber in January 5th, 2021 due to “fever accompanied with cough for more than 10 days.” Novel Coronavirus nucleic acid test was negative. The patient developed fever after catching a cold more than 10 days before admission, with the temperature fluctuating between 37 and 38°C, accompanied by cough, fatigue, chills, and pharyngeal discomfort. The symptoms were not relieved even after “GanKang” was administered. The symptoms aggravated in December 5th, 2020, with the temperature peaking to 41°C and reducing to below 38°C after ibuprofen was administered orally. The hospitalization inspection at the local hospital showed the following: WBC 3.3 × 10^9^/L, Hb 85 g/L, PLT 87 × 10^9^/L. Bone marrow puncture indicated that hematopoietic tissue hyperplasia was active and the proportion of three-line blood cells was roughly normal. Lymphocytes accounted for 9.5%, and atypical lymphocytes were found occasionally. Adenovirus IgM was weakly positive, ferritin: >2,000 ng/ml, and procalcitonin: 4.14 ng/ml. Abdominal ultrasound and CT scan indicated hepatomegaly and splenomegaly. Cerebrospinal fluid: glucose 3.8 mmol/L, chlorine 125 mmol/L, protein 0.28 g/L, and nucleated cells 3 × 10^6^/L. The respiratory tract had 13 types of virus and complete immunophenotyping of leukemia; echocardiography showed no evident abnormalities. After treatment with cefoperazone sulbactam sodium, the symptoms were not significantly relieved, and the diagnosis was not clear, so the patient came to our hospital for further treatment. Vital signs: T: 37.1°C, P: 110/min, R: 19/min, BP: 110/72 mmHg. Hospitalization inspection after admission: Blood routine examination: WBC 4.68 × 10^9^/L, Hb 100 g/L, and RBC3.65 × 10^12^/L. Biochemical test: ALT 204 IU/L; AST 176 IU/L; GGT 153 IU/L; GLU tendency for 6.66/L; TG tendency for 2.29/L; 0.30 tendency for HDL – C/L; LDH 889 IU/L; Ferritin: >2,000 ng/ml; PCT: 2.11 ng/ml; CRP: 53.5 mg/L, IL-6 31.58 pg/ml. TORCH + EBV: CMV, HSV, rubella virus, and EBV IgG were all positive. Coombs' test (direct anti-human globulin test): positive, ++. Autoimmune antibody: ANA + 1:100; Anti-jo-1 antibody +; RF 207 IU/ml; HIV, and syphilis tests were negative. Blood culture: no bacterial growth was observed in 5 days. T cell subsets: (–). Chest CT showed no obvious abnormality. Abdominal ultrasound showed that the spleen thickness was ~6.6 cm intercostal, suggesting splenomegaly (Figure 3A). The diagnosis was considered to be: (1) FUO:sepsis with hematological disease? Do not exclude the possibility of parasitic diseases? Hemophilic cell syndrome? Lymphoma? Autoimmune disease? (2) Mild anemia. (3) Splenomegaly. The cause of the fever was unclear, and an mNGS test was performed on the second day after admission to the emergency department. Emergent treatment: Ceftriaxone 2.0ivgtt Qd for 3 days and dexamethasone administered to reduce the fever; however, the patient showed no significant relief of fever. On the third day, the patient was admitted to the department of infectious diseases. On the fourth day, the mNGS test results of the patient showed *Leishmania infantum* (Figure 4). CDC was contacted for rK39 antibody test, and the antibody was detected to be positive on the seventh day, which is in accordance with the criteria for clinically diagnosed cases (10). The anti-kala-azar treatment was started and sodium stibogluconate was administered. The patient was given 600 mg SSG daily for 10 consecutive days intravenously, and the drug was discontinued after the patient's symptoms improved significantly. The patient's temperature began to gradually return to normal 3 day later. On the 17th day, the temperature was normal. His spleen retracted, and he was discharged after bone marrow puncture. The patient underwent 3 bone marrow puncture examinations successively. On the 11th day of admission, hematopoietic cells in the bone marrow were actively hyperplasia, and the immunohistochemistry showed scattered lymphocytes and a few suspicious EBER positive cells. On the 17th day, the bone marrow hematopoietic cells were hyperactive. On the 14th day of admission, the spleen was retracted, and the thickness of the spleen was about 5.1 cm (Figure 3B). One month after discharge, the follow-up showed that the bone marrow hematopoietic cell hyperplasia was low. The intercostal spleen thickness was ~4.5 cm (Figure 3C). The blood routine and liver function returned to normal.

## Discussion

Kala-azar is one of the most neglected infectious diseases globally, with ~1.5–2 million new cases reported every year. It is endemic in more than 60 countries worldwide, such as the Mediterranean, Middle East, sub-Saharan Africa, southern Europe, India, Pakistan, and China. As its prevention and control were carried out in China, kala-azar has been nearly eliminated, but there is a small-range prevalence in Xinjiang, Gansu, Sichuan, Shaanxi, and Shanxi (11). The first patient, in this case, had a history of epidemic areas in Africa. Therefore, it interfered with the doctors' inquiry about other epidemics and ignored other infectious diseases. The second case was a case who lived in Chongqing city and had a history of Yangquan, Shanxi Province residence. The lack of knowledge of this rare infectious disease among front-line clinicians and the atypical clinical manifestations of kala-azar led to the easy misdiagnosis and missed diagnosis of kala-azar. In addition to the strengthening knowledge dissemination and training on infectious diseases, strengthening clinical etiology detection technology is also one of the main ways to improve the level of diagnosis and treatment of infectious diseases.

The clinical manifestations of kala-azar are complex, and the symptoms overlap with those of severe infections, hematologic diseases, and autoimmune diseases, such as fever, splenomegaly, and cytopenia, which cause great confusion to doctors and are not easy to identify. Kala-azar can hardly achieve self-healing, and the mortality rate can exceed 90% if the treatment is not accepted (2). Clinical studies have reported that the misdiagnosis rate of kala-azar is 84.2% (12, 13). The misdiagnosis might be due to the lack of clinical awareness and attention to kala-azar and insufficient epidemiological history data. Kala-azar is easily misdiagnosed as connective tissue disease, especially systemic lupus erythematosus (14). The positive rates of ANA and RF in kala-azar are 88 and 63%, respectively (15). The immunologic tests of the two patients in this study were as follows: Coombs' test: positive, ++; ANA +1:100; Anti-jo-1 antibody +; and RF 207 IU/ml. These results are consistent with those obtained previously (16). The polyclonal activation of kala-azar B cells is considered the main cause of immunoglobulin and antibody formation (17).

The detection of the kala-azar pathogen is difficult. The clinical diagnosis of kala-azar is mainly achieved by bone marrow smear, histopathological biopsy etiology, and rK39 immunochromatographic strip test, among which the discovery of *Leishmania* parasite in bone marrow, lymph nodes, spleen, skin, and other tissues is considered the golden criterion of diagnosis. The positive rate of bone marrow puncture is 53–86% (1). Lymph node biopsy has a sensitivity of 65.1% for kala-azar diagnosis (18). The first patient in this study had low platelet count, abnormal coagulation function, and high puncture risk, which limited the bone marrow puncture examination. In the second patient, all three attempts of bone marrow punctures were failed to diagnose *Leishman-Donovan* body, which increased the difficulty in the diagnosis. The detection rate of pathogens in splenic puncture smear is high (19), and the risk of splenic puncture is also relatively high. The positive rate of the rK39 immunochromatographic strip test for detecting leishmaniasis antibodies was high, and according to the diagnostic criteria of leishmaniasis, although the suspected cases + immunological test results were clinically diagnosed cases of leishmaniasis, and not confirmed cases, they could still guide clinical treatment. Hospitals that do not have access to rK39 test should contact their local CDC for further testing (3). Various PCR-based detection methods have shown good application prospects, but currently there is a lack of applicable commercial kits in China, and molecular diagnosis has not been widely carried out (6). At present, the emergence and development of second-generation sequencing technology provide convenient diagnoses for rare pathogens. McCarthy et al. (20) used mNGS for the first time to investigate the populations of *Lutzomyia longipalpis* associated with visceral leishmaniasis, which helps to monitor the occurrence of insect vector-borne infectious diseases. There are a few reports on the detection of *Leishmania* by mNGS in China. In this study, mNGS testing was performed in both patients at an early stage in the emergency department, thus enabling rapid diagnosis. The first patient had a poor prognosis due to time-consuming out-of-hospital diagnosis. The second patient benefited from rapid and accurate early diagnosis in an emergency department and had a good prognosis.

This case study does have some limitations: (1) As this study is a case report, the leishmania was detected by mNGS in two cases, which is not generally representative. The significance of mNGS in the detection and diagnosis of acute infectious diseases needs to be further studied. (2) The rK39 antibody test is highly sensitive to kala-azar diagnosis, but it is not a routine test item for the requisition of CDC application, which limits its clinical use in medical institutions and makes the universal screening of kala-azar unachievable.

Currently, the clinical diagnosis of rare infectious diseases, such as kala-azar, is relatively difficult. The metagenomics sequencing technology, which is a second-generation sequencing technology, has been gradually applied to the pathogen detection of clinical infectious diseases owing to its advantages of being unbiased, fast, and accurate and having wide coverage. Moreover, it has important clinical value for suspicious and complicated infectious diseases. It is helpful for emergency doctors to diagnose and treat this special pathogen early, to advance patient treatment and improve their prognosis. In short, kala-azar requires early stage diagnosis and treatment to achieve a relatively satisfactory prognosis. The mNGS detection is beneficial to the diagnosis and treatment of infectious diseases with unknown causes in the early stage of emergency treatment.

## Data Availability Statement

The datasets presented in this study can be found in online repositories. The names of the repository/repositories and accession number(s) can be found below: NCBI - PRJNA814613.

## Ethics Statement

The studies involving human participants were reviewed and approved by Ethical Committee of West China Hospital, Sichuan University. Written informed consent to participate in this study was provided by the participants' legal guardian/next of kin. Written informed consent was obtained from the individual(s), and minor(s)' legal guardian/next of kin, for the publication of any potentially identifiable images or data included in this article.

## Author Contributions

HG: writing—original draft preparation. TL and JW: writing—review and editing. SZ: idea. All authors read and approved the final manuscript.

## Conflict of Interest

The authors declare that the research was conducted in the absence of any commercial or financial relationships that could be construed as a potential conflict of interest.

## Publisher's Note

All claims expressed in this article are solely those of the authors and do not necessarily represent those of their affiliated organizations, or those of the publisher, the editors and the reviewers. Any product that may be evaluated in this article, or claim that may be made by its manufacturer, is not guaranteed or endorsed by the publisher.
